# Atrial and ventricular cardiomyopathy associated with premature atrial contractions: Speckle-tracking echocardiography demonstrates reversibility following successful ablation

**DOI:** 10.1016/j.hrcr.2022.01.001

**Published:** 2022-01-10

**Authors:** Satoshi Higuchi, Eun-Jeong Kim, Edward P. Gerstenfeld, Dwight Bibby, Nelson B. Schiller, Henry H. Hsia

**Affiliations:** ∗ Division of Cardiology, Section of Cardiac Electrophysiology, University of California, San Francisco, San Francisco, California; † Research Cardiac Physiology Laboratory, University of California, San Francisco, San Francisco, California

**Keywords:** Premature atrial complex, Atrial myopathy, Cardiomyopathy, Remodeling, Catheter ablation

**Key Teaching Points**

• Frequent premature atrial complexes (PACs) might cause a secondary cardiomyopathy not only in the left ventricle but also in the left atrium. However, both atrial and ventricular myopathy can be reversed with the elimination of the PACs.
• Two-dimensional speckle-tracking strain imaging would allow quantitative evaluation of PAC-induced cardiomyopathy and dyssynchrony.
• Intra- and/or interventricular ventricular dyssynchrony due to aberrant conduction may be associated with the pathophysiologic mechanism in the cardiomyopathic process, similar to the role of PVCs in arrhythmia-induced cardiomyopathy.

## Introduction

Premature ventricular complex (PVC)-induced cardiomyopathy has been recognized as a reversible form of left ventricle (LV) systolic dysfunction associated with frequent ventricular ectopy.^1^, ^2^, ^3^ However, a reversible impairment of LV systolic function secondary to frequent premature atrial complex (PAC) exposure has rarely been encountered and therefore, the entity of PAC-induced cardiomyopathy remains to be elucidated. In the present case, we demonstrated clear evidence of both atrial and ventricular cardiomyopathies caused by frequent PACs using 2-dimensional speckle-tracking strain imaging.

## Case report

**Figure 1:**
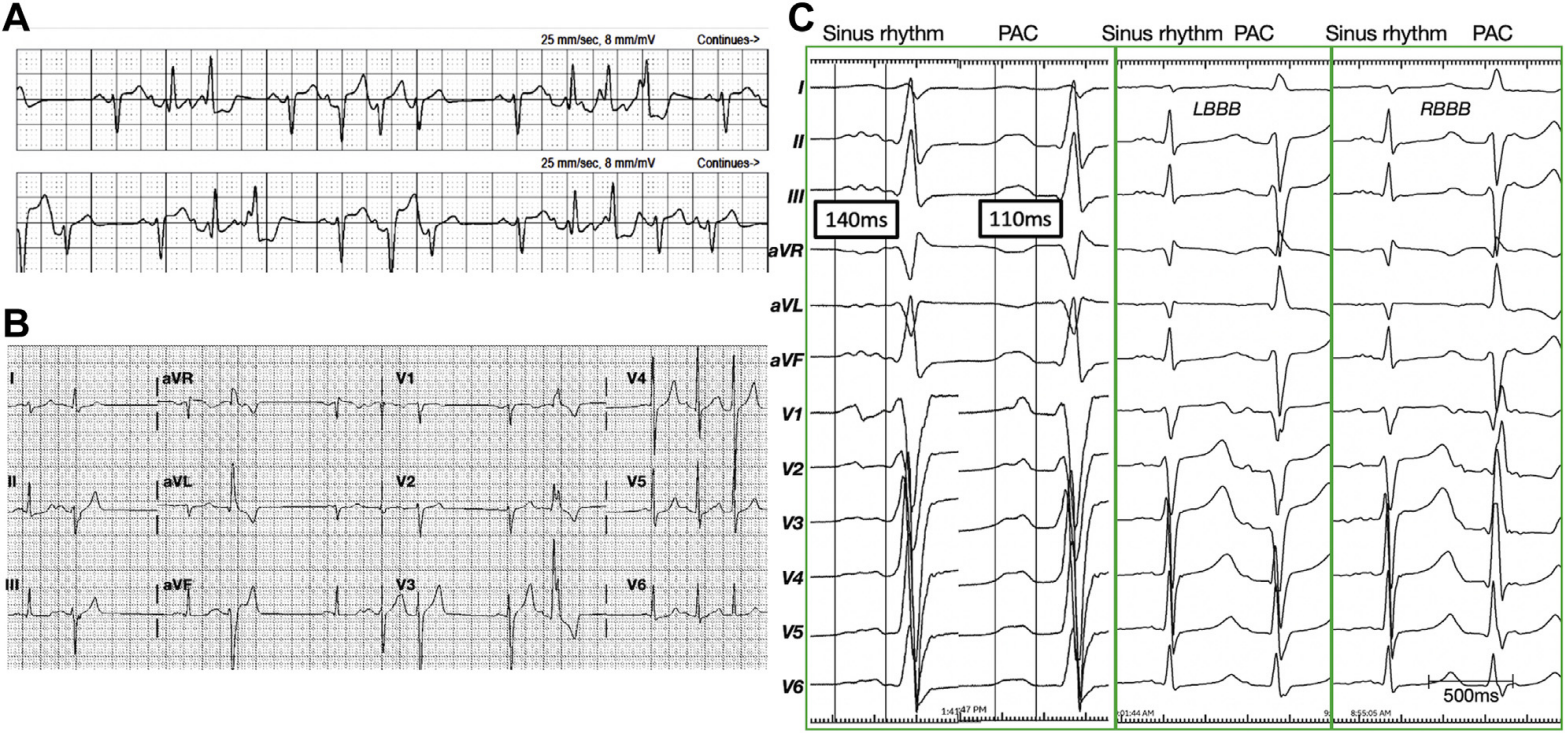
Electrocardiograms (ECG) of the ectopic beats. **A:** Monitored ECG 2 years prior to patient’s visit. **B, C:** Twelve-lead ECG showing sinus rhythm with ectopic beats (**B**) and premature atrial complexes (PAC; **C**) with or without different degrees of aberrancy.

A 35-year-old man initially presented with a nonischemic cardiomyopathy, severe LV dysfunction, and an ejection fraction of less than 20% in 2019. He had a history of palpitations since a teenager without any other significant past medical history or family history. Cardiac monitoring revealed a high burden of PVCs (17%) and nonsustained ventricular tachycardia (Figure 1A). His 12-lead electrocardiogram exhibited frequent ectopic beats with narrow and wide QRS complexes (Figure 1B). Coronary angiography and cardiac magnetic resonance imaging demonstrated no evidence of myocardial scar/fibrosis. He was treated with guideline-directed medical therapy for heart failure that included β-blocker, angiotensin receptor–neprilysin inhibitor, and digoxin. A recent transthoracic echocardiogram using a Vivid IQ (GE Healthcare, Milwaukee, WI) exhibited a severely dilated LV (end-diastolic volume index 109 mL/m^2^ and end-systolic volume index 65 mL/m^2^) and enlarged left atrium (LA) (volume index 43 mL/m^2^). The biplane Simpson’s measurement exhibited a global reduced left ventricular ejection fraction (LVEF) of 41% (Supplemental Figure 1A).

**Figure 2:**
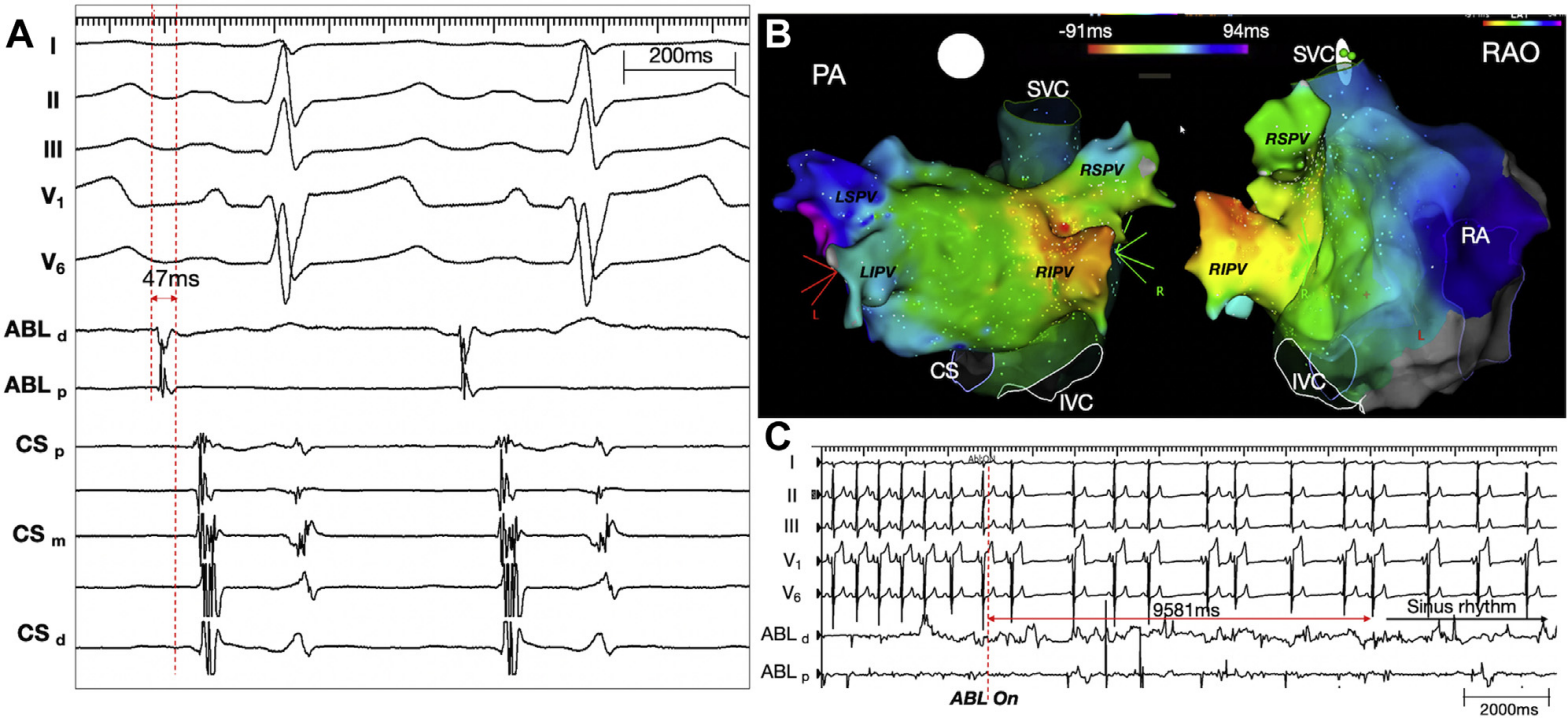
Catheter ablation of premature atrial complexes (PACs). **A:** Intracardiac electrogram showing the earliest local atrial signals during short runs of PACs. **B:** The earliest site is noted in the right inferior pulmonary vein (RIPV) near the carina (*red spot*). **C:** A radiofrequency application at the earliest site resulted in the elimination of all PACs. Subscripts d, m, and p indicate the distal, mid, and proximal electrode pairs of the relevant catheter. ABL = ablation catheter; CS = coronary sinus; IVC = Inferior vena cava, LIPV = left inferior pulmonary vein; LSPV = left superior pulmonary vein; PA = posterior-anterior; RA = right atrium; RAO = right anterior oblique; RSPV = right superior pulmonary vein; SVC = superior vena cava.

The patient was originally referred for catheter ablation of frequent PVCs. However, upon careful inspection, the variable ectopic beats appeared to be PACs with a variable degree of aberrancy (Figure 1B and 1C). The P-wave morphology during the PACs was narrower than that of the sinus P waves and was positive in lead I and the inferior leads, and biphasic (iso/pos) in lead V_1_ (Figure 1C). These features suggested a left atrial septal origin. During activation mapping, the earliest atrial electrogram preceded the P-wave onset by 47 ms (Figure 2A) and was localized to the right inferior pulmonary vein near the carina (red spot in Figure 2B). Focal radiofrequency ablation in that region resulted in the elimination of all atrial ectopies within 9.6 seconds (Figure 2C).

**Figure 3:**
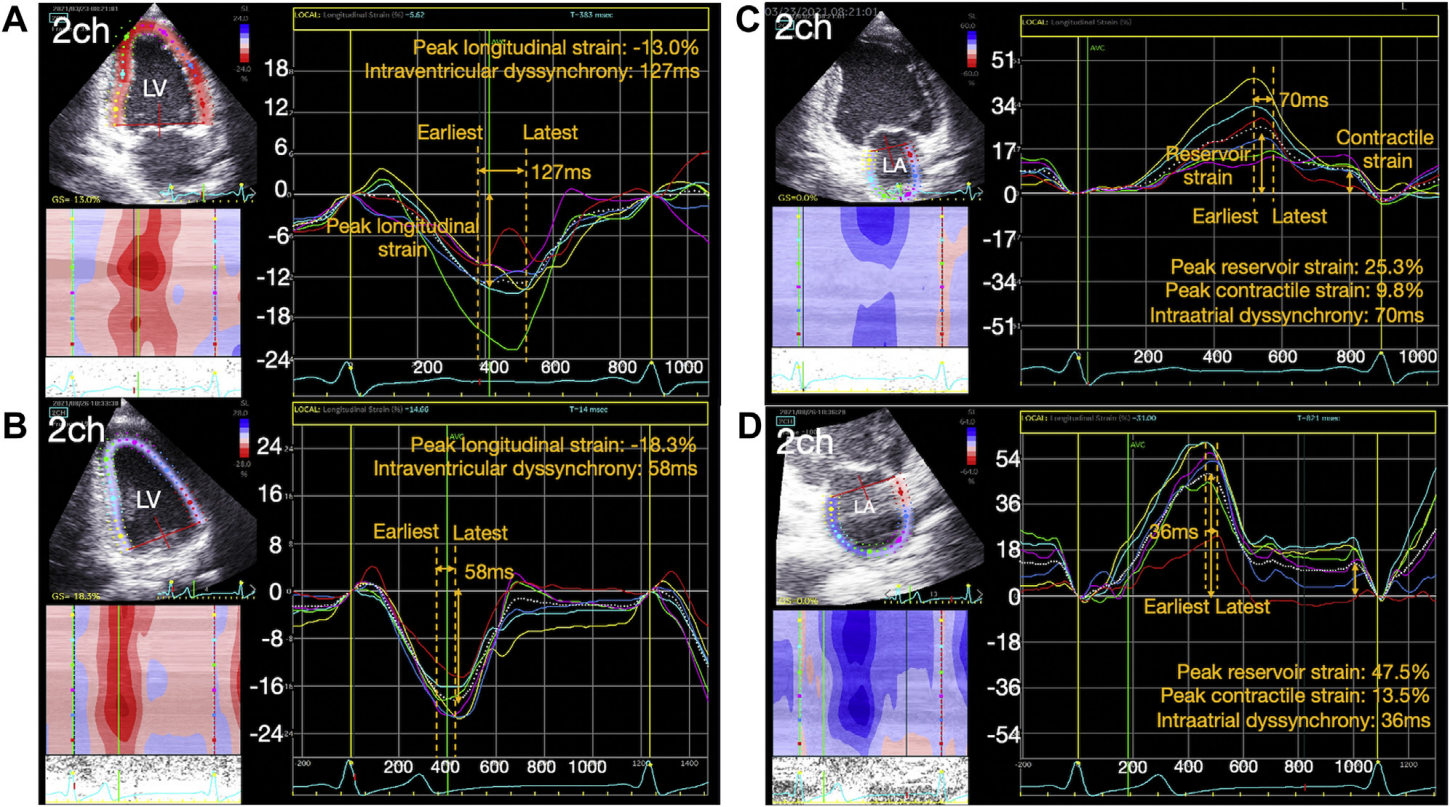
Difference in the left ventricle (LV) and left atrium (LA) function using speckle-tracking strain imaging before and 5 months after ablation. **A, B:** Comparisons of the LV function quantified by 2-dimensional (2D) speckle-tracking imaging (EchoPAC; GE Healthcare, Horten, Norway) between that before (**A**) and that 5 months after the PAC ablation (**B**), which was obtained from the apical 2-chamber view. The global LV longitudinal strain was assessed by measuring the average of the peak longitudinal strain across 18 segments obtained from the apical 2-chamber, 3-chamber, and 4-chamber views, respectively, at a frame rate of ≥70 frames per second. The intraventricular dyssynchrony was defined as the difference in the time-to-peak of the earliest and latest activated segments among the 6 segments for each view. **C, D:** Comparisons of the LA function quantified by 2D speckle-tracking imaging between that before (**C**) and that 5 months after the PAC ablation (**D**), which was obtained from the apical 2-chamber view. The global LA reservoir strain, contractile strain (averaged across 12 segments: apical 4- and 2-chamber views), and intra-atrial dyssynchrony were obtained by the same methods used for the LV strain. To measure the strain values, the QRS onset of the electrocardiogram was used as a reference point. See Supplemental Figure 2 for the remaining views of the LV and LA images.

After the procedure, the patient experienced resolution of his palpitations with an improved exercise capacity. Five months later, a repeat Holter monitoring documented only rare isolated PACs. A follow-up transthoracic echocardiogram with 2-dimensional speckle-tracking strain imaging using an EchoPAC workstation (GE Healthcare, Horten, Norway) demonstrated a marked improvement in the size and function of both the LA (volume index 26 mL/m^2^) and LV (end-diastolic volume index 64 mL/m^2^, end-systolic volume index 31 mL/m^2^, and an improved LVEF of 52%) (Supplemental Figure 1B). The LV longitudinal deformation, which was quantified by measuring the global longitudinal strain, improved from -13.6% to -18.2% (Figure 3 and Supplemental Figure 2A and 2B). The global peak atrial reservoir strain as well as contractile strain also improved, from 29.8% to 48.2% and from 9.3% to 14.1%, respectively (Figure 3 and Supplemental Figure 2C and 2D). Finally, the marked heterogeneity in the regional LA and LV strain improved after the PAC ablation, implying a synchronous coordination in both chambers (Figure 3 and Supplemental Figure 2). All heart failure medications were discontinued and the patient remains completely asymptomatic without exertional limitation.

## Discussion

We demonstrated a unique case of a PAC-induced cardiomyopathy, characterized by reversible chamber dilation and dysfunction associated with a high burden of PACs. The present case highlighted several novel findings. To the best of our knowledge, this was the first case that provided a quantitative evaluation of the cardiac function in an atrial ectopy–induced cardiomyopathy. Although a speckle-tracking analysis requires a certain level of operator skill to achieve a consistent image quality, previous reports had demonstrated that speckle-tracking echocardiography was able to detect subtle and early forms of cardiomyopathy associated with frequent PVCs, even among those who had a normal LVEF.^4^^,^^5^ Our case demonstrated that such quantitative assessments of contraction synchrony as well as longitudinal shortening are useful in early detection and risk stratification even in a case of a PAC-induced cardiomyopathy.

Although population-based cohort studies have demonstrated an association between frequent PACs and atrial myopathy,^6^ the causal relationship between frequent PACs and the development of an atrial myopathy is unknown. We found clear evidence of a secondary atrial myopathy and regional incoordination using detailed speckle-tracking echocardiography, and such an atrial myopathy was also completely reversible upon a successful PAC ablation. Therefore, this finding provides insight into the role of an arrhythmia-related regional incoordination, which may lead to adverse atrial remodeling, abnormal substrate development with the risk of atrial fibrillation, and a stroke.^7^^,^^8^

There have been a few small case series that have reported LV cardiomyopathy secondary to frequent PACs.^9^, ^10^, ^11^, ^12^, ^13^ The mechanism of PAC-induced ventricular cardiomyopathy remains poorly defined and prior studies in animal models have shown controversial results.^2^^,^^14^^,^^15^ All reported cases of PAC-induced cardiomyopathy have shown a high PAC burden, between 19% and 40% (median 21%), and in most cases the PACs have been present and symptomatic for many years before the cardiomyopathy was recognized.^9^, ^10^, ^11^, ^12^, ^13^ Our case supported the notion that the frequency and duration of the PACs are important in contributing to atrial and ventricular cardiomyopathy. All patient cohorts were in their third or fourth decade of life (aged 23–44; median age: 33 years). Therefore, an age-related sensitivity or genetic factors may also play an important role in the pathophysiology of PAC-induced cardiomyopathy.

In this case, frequent PACs were associated with a variable aberrant conduction. Intra- and/or interventricular ventricular dyssynchrony due to conduction disturbances may be an important pathophysiologic mechanism in the development of the cardiomyopathic process, similar to the role of PVCs in arrhythmia-induced cardiomyopathy.^2^ The combination of dyssynchrony and arrhythmia irregularity, as well as unfavorable coupling intervals, may cause an abnormal myocardial activation and contractile patterns leading to a constellation of secondary hemodynamic, electrical, structural, and neurohormonal changes causing cardiomyopathy to develop.^2^^,^^3^ By using speckle-tracking echocardiography, this case provided pathophysiologic insight into the potential mechanisms of both arrhythmia-induced atrial and ventricular cardiomyopathies.

## Conclusion

PACs are usually considered to be benign. However, our case highlighted that a high burden of PACs can lead to a secondary cardiomyopathy in the LA and/or LV, which is reversible with the elimination of the PACs with ablation. A careful surveillance of patients with a high burden of PACs is warranted, and an echocardiogram with speckle tracking may aid in an earlier detection of cardiomyopathy development.
