# Supplementary material for: Atrial and ventricular cardiomyopathy associated with premature atrial contractions: Speckle-tracking echocardiography demonstrates reversibility following successful ablation
Source: HeartRhythm Case Rep. 2022 Jan 10;8(4):243–6. doi: 10.1016/j.hrcr.2022.01.001 (PMC9039110; doi:10.1016/j.hrcr.2022.01.001)
Supplement: Supplemental Figures [file mmc1.pdf]

SUPPLEMENTAL MATERIAL

Figure legends:

Supplemental Figure 1. Transthoracic echocardiography before and 5 months after ablation

Echocardiographic images of the LV and LA from the apical 4-chamber view **(A)** before and **(B)** 5 months after the ablation of the PACs. LVEDVI = left ventricular end-diastolic volume index, LVESVI = left ventricular end-systolic volume index, EF = ejection fraction, MR = mitral regurgitation, LAVI = left atrial volume index, LV = left ventricle, LA = left atrium.

Supplemental Figure 2. Difference in the LV function (apical 3-chamber and 4-chamber) and LA function (apical 4-chamber) using speckle tracking strain imaging before and 5 months after ablation

Comparisons of the LV function quantified by 2D speckle tracking imaging (Echo-PAC) between **(A)** that before and **(B)** that 5 months after the PAC ablation, which was obtained from the apical 3- and 4-chamber views. **(C)** The LA function quantified by 2D speckle tracking imaging before ablation and **(D)** that 5 months after the ablation, which was obtained from the apical 4-chamber view. All measurements of the longitudinal strain and intraventricular/atrial dyssynchrony were obtained by the same methods used in Figure 3.

Supplemental Figure 1. Transthoracic echocardiography before and 5 months after ablation

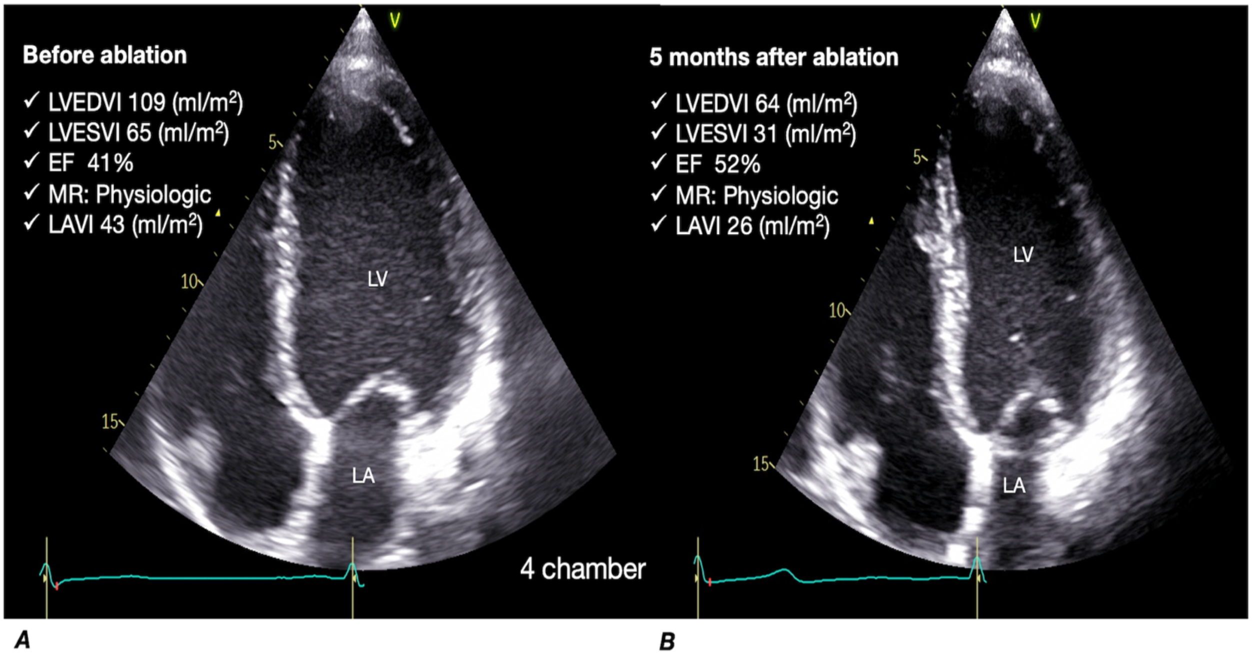

Echocardiographic images of the LV and LA from the apical 4-chamber view **(A)** before and **(B)** 5 months after the ablation of the PACs. LVEDVI = left ventricular end-diastolic volume index, LVESVI = left ventricular end-systolic volume index, EF = ejection fraction, MR = mitral regurgitation, LAVI = left atrial volume index, LV = left ventricle, LA = left atrium.

Supplemental Figure 2. Difference in the LV function (apical 3-chamber and 4-chamber) and LA function (apical 4-chamber) using speckle tracking strain imaging before and 5 months after ablation

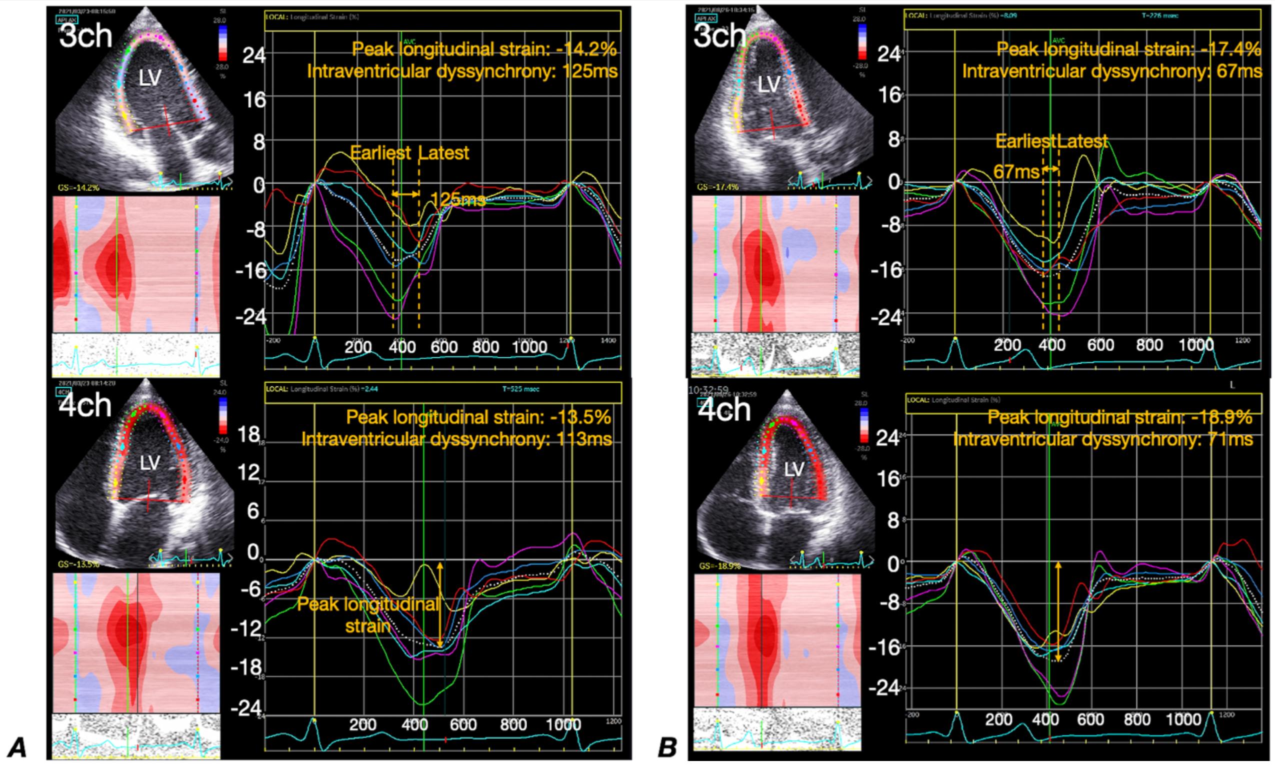

Comparisons of the LV function quantified by 2D speckle tracking imaging (Echo-PAC) between **(A)** that before and **(B)** that 5 months after the PAC ablation, which was obtained from the apical 3- and 4-chamber views.

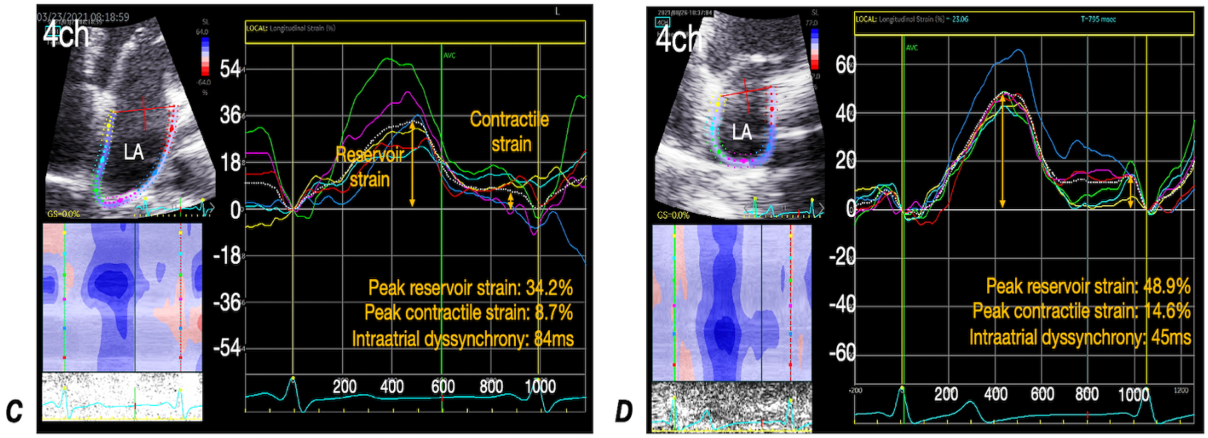

(C) The LA function quantified by 2D speckle tracking imaging before ablation and **(D)** that 5 months after the ablation, which was obtained from the apical 4-chamber view. All measurements of the longitudinal strain and intraventricular/atrial dyssynchrony were obtained by the same methods used in Figure 3.
